# Genome Sequencing Illustrates the Genetic Basis of the Pharmacological Properties of *Gloeostereum incarnatum*

**DOI:** 10.3390/genes10030188

**Published:** 2019-03-01

**Authors:** Xinxin Wang, Jingyu Peng, Lei Sun, Gregory Bonito, Jie Wang, Weijie Cui, Yongping Fu, Yu Li

**Affiliations:** 1 Engineering Research Center of Chinese Ministry of Education for Edible and Medicinal Fungi, Jilin Agricultural University, Changchun 130118, China; wangx220@msu.edu (X.W.); sunlei@jlau.edu.cn (L.S.); cuiweijie825@126.com (W.C.); 2 Department of Plant Protection, Shenyang Agricultural University, Shenyang 110866, China; 3 Department of Plant, Soil, and Microbial Sciences, Michigan State University, East Lansing, MI, USA; pengjin2@msu.edu (J.P.); bonito@msu.edu (G.B.); 4 Department of Plant Biology and Center for Genomics Enabled Plant Science, Michigan State University, East Lansing, MI, USA; wangjie6@msu.edu

**Keywords:** *Gloeostereum incarnatum*, whole genome sequencing, PacBio, secondary metabolite, cytochrome P450 enzyme (CYP), terpenoid

## Abstract

*Gloeostereum incarnatum* is a precious edible mushroom that is widely grown in Asia and known for its useful medicinal properties. Here, we present a high-quality genome of *G. incarnatum* using the single-molecule real-time (SMRT) sequencing platform. The *G. incarnatum* genome, which is the first complete genome to be sequenced in the family *Cyphellaceae*, was 38.67 Mbp, with an N50 of 3.5 Mbp, encoding 15,251 proteins. Based on our phylogenetic analysis, the *Cyphellaceae* diverged ~174 million years ago. Several genes and gene clusters associated with lignocellulose degradation, secondary metabolites, and polysaccharide biosynthesis were identified in *G. incarnatum*, and compared with other medicinal mushrooms. In particular, we identified two terpenoid-associated gene clusters, each containing a gene encoding a sesterterpenoid synthase adjacent to a gene encoding a cytochrome P450 enzyme. These clusters might participate in the biosynthesis of incarnal, a known bioactive sesterterpenoid produced by *G. incarnatum*. Through a transcriptomic analysis comparing the *G. incarnatum* mycelium and fruiting body, we also demonstrated that the genes associated with terpenoid biosynthesis were generally upregulated in the mycelium, while those associated with polysaccharide biosynthesis were generally upregulated in the fruiting body. This study provides insights into the genetic basis of the medicinal properties of *G. incarnatum*, laying a framework for future characterization of bioactive proteins and pharmaceutical uses of this fungus.

## 1. Introduction

Mushrooms are an important source of nutrition, and a growing body of evidence has indicated that mushrooms may have medicinal properties and human health benefits [1,2,3]. *Gloeostereum incarnatum* (family Cyphellaceae) is an edible mushroom, which grows as a saprophyte on broad-leaved trees [4]. *G. incarnatum* is native to China, but is popular in other regions in Asia too, such as Japan and Siberia [4]. Besides its savory taste, *G. incarnatum* is well-known for its medicinal properties. Antioxidant, immunomodulatory, anti-inflammatory, anti-proliferative, and antibacterial properties have been attributed to this mushroom [5,6,7]. Recent studies have shown that sesquiterpenes and polysaccharides are the main bioactive compounds underlying the beneficial effects of *G. incarnatum* [5,6,8].

With the rapid advancement of sequencing technologies, the number of available fungal genomes has increased [9,10]. However, genomes of medicinal mushrooms remain scarce. Recently, the genomes of a few medicinal mushrooms (e.g., *Ganoderma lucidum*, *Antrodia cinnamomea*, and *Hericium erinaceus*) were released, and proteins putatively associated with the pharmacological properties of these mushrooms were investigated [11,12,13]. Gene clusters associated with the synthesis of various bioactive secondary metabolites, such as terpenoids and polypeptides, have been identified in many medical mushroom genomes [11,13]. For instance, nine gene clusters associated with the cytochrome P450 (CYP) and triterpenoid pathways were identified in *A. cinnamomea* [11], while four gene clusters associated with terpene and polyketide biosynthesis were identified in *H. erinaceus* [13]. In *G. lucidum*, 24 physical CYP gene clusters, possibly involved in triterpenoid biosynthesis, were identified [12]. Although several bioactive compounds have been identified in *G. incarnatum* [5,6,8,14], the genetic basis of the medicinal benefits of this mushroom are largely unknown.

In this study, we used the Pacific Biosciences (PacBio) long-read sequencing platform [15] to perform the de novo assembly of the *G. incarnatum* genome. This is the first genome to be sequenced in the Cyphellaceae family. We also compared the transcriptome profiles of the mycelium and the fruiting body, the two major developmental stages of *G. incarnatum*. The sequenced genome of *G. incarnatum* presented herein is, to our knowledge, one of the most comprehensive assembled genomes of an edible mushroom. In this study, we aimed to (1) present a high-quality reference genome for *G. incarnatum*, which can be used for future analyses of genome function and genetic variation and (2) identify relevant functional genes, gene clusters, and signaling pathways associated with the saprophytic lifestyle and pharmaceutical properties of *G. incarnatum*. We specifically focused on terpene biosynthesis, cytochrome P450 enzyme biosynthesis, and polysaccharide production. Our study provides a valuable genomic and transcriptomic resource for future studies of the genetic basis of the medicinal properties of *G. incarnatum*. Such studies would represent a first step towards realizing the full potential of *G. incarnatum* as a source of pharmacologically active compounds on an industrial scale.

## 2. Materials and Methods

### 2.1. Fungal Material, Sequencing, and Genome Assembly

We isolated protoplast-derived monokaryons from the dikaryotic strain of the *G. incarnatum* commercial strain CCMJ2665. The monokaryons were obtained as described previously [16], except that the dikaryotic mycelia were incubated for 240 min at 30 °C in lywallzyme lysing enzyme. The single-nucleated genomic DNA of the *G. incarnatum* monokaryon strain was then used for genome sequencing and annotation. Genomic DNA was extracted using NuClean Plant Genomic DNA Kits (CWBIO, Beijing, China). The genome of *G. incarnatum* was sequenced on a PacBio Sequel long-read sequencing platform with a library insert size of 20 kb, at the Engineering Research Center of the Chinese Ministry of Education for Edible and Medicinal Fungi, Jilin Agricultural University (Changchun, China). Raw data were assembled with SMARTdenovo (https://github.com/ruanjue/smartdenovo). The completeness of the genome assembly was evaluated using Core Eukaryotic Genes Mapping Approach (CEGMA) [17] and Benchmarking Universal Single-Copy Orthologs (BUSCO; [18]). The whole-genome sequence of *G. incarnatum* has been deposited in GenBank (in submission). The genome reported in this study has been deposited in GenBank under the accession RZIO00000000.

### 2.2. Genome Annotation

Three different strategies were used to predict genes in the *G. incarnatum* genome: Sequence homologies with four representative mushrooms; ab initio with Augustus [19], Genescan [20], GlimmerHMM [21], and SNAP [22]; and combining extrinsic and ab initio approaches with GLEAN (http://sourceforge.net/projects/glean-gene). GLEAN gene prediction results were used for subsequent analyses. Protein-coding genes were annotated by GLEAN using both ab initio and evidence-based methods [23]. Predicted genes were functionally annotated against several databases—National Center for Biotechnology Information (NCBI) non-redundant (nr), Swiss-Prot, and InterPro—using BLASTP searches (e-value ≤ 1 × 10^−5^). Gene annotations were refined using the following databases: Gene Ontology (GO) [24], Clusters of Orthologous Groups (KOG) [25], and Kyoto Encyclopedia of Genes and Genomes (KEGG) [26]. Transposon sequences were identified by aligning the assembled genome to the Repbase database [27] with RepeatMasker (version 3.3.0; http://www.repeatmasker.org/; [28]) and RepeatProteinMasker [22]. Tandem repeat sequences (TRF) were predicted with Tandem Repeat Finder [29]. Ribosomal RNA (rRNA) sequences were identified, based on sequence homology and also through use of de novo prediction strategies with rRNAmmer [30]. Transfer RNA (tRNA) genes were identified using tRNAscan-SE [31]. Non-coding RNAs, such as small nuclear RNA (snRNAs) and microRNAs (miRNAs), were predicted with Rfam [32].

### 2.3. Evolutionary Analysis and Phylogeny

The phylogenetic analysis was performed using single-copy genes shared across *G. incarnatum* and another nine fungal species (*Omphalotus olearius*, *Gymnopus luxurians*, *Laccaria bicolor*, *Coprinopsis cinerea*, *Armillaria ostoyae*, *Lentinula edodes*, *Schizophyllum commune*, *Serpula lacrymans* and *Coniophora puteana*). The “all against all” BLASTP searches were performed with a cutoff e-value of 1 × 10^−7^ for proteins from all species. The alignments of gene pairs were conjoined by solar. Only gene pairs with an alignment ratio (aligned region by total length) of more than 30% in both homologous genes were kept for the following gene family construction. Gene families were clustered using a sparse graph of gene relationships using the hierarchical clustering algorithm hcluster_sg 0.5.1 package. Finally, we identified single-copy genes which had only one homolog per taxon, and those genes were used to construct the phylogenetic tree. The protein sequences of these single-copy genes were aligned using MUSCLE [33] and the protein alignments were transformed into codon alignments with PAL2NAL. Gblocks was used to refine each codon alignment, and all refined alignments were concatenated to a super codon alignment. RAxML software (version 7.2.3) [34] was used to construct the phylogenetic tree using the maximum likelihood (ML) algorithm. The best-scoring ML tree was inferred using the rapid bootstrap analysis after 1000 runs. The divergence times among species were estimated using the mcmctree module in PAML [35] with the calibration time of *Serpula lacrymans* and *Coniophora puteana* according to Floudas et al. (2012).

### 2.4. Carbohydrate-Active Enzyme (CAZyme) Family Classification

The CAZymes in the *G. incarnatum* genome were identified by mapping the annotated protein sequences to the CAZy database (http://www.cazy.org/) [36] using BLASTP (cut-off e-value ≤ 1 × 10^−5^, identity ≥ 40% and coverage ≥ 40%). The recovered CAZymes were classified as glycoside hydrolases (GHs), auxiliary activities (AAs), carbohydrate-binding modules (CBMs), glycosyl transferases (GTs), polysaccharide lyases (PLs), and carbohydrate esterases (CEs).

### 2.5. Cytochrome P450 (CYP) Predictions

CYP proteins were predicted by aligning the gene models to the fungal P450 database (http://p450.riceblast.snu.ac.kr/index.php?a=view;) with BLASTP (e-value ≤ 1 × 10^−5^, matrix = BLOSUM62). CYP proteins were assigned to protein families based on Nelson’s nomenclature [37]. For protein sequences that aligned with multiple families, the top hit was chosen.

### 2.6. Secondary Metabolite Annotations

Secondary metabolite gene clusters were predicted with fungal AntiSMASH 3.0 (https://fungismash.secondarymetabolites.org/) [38], with the default parameter values.

### 2.7. RNA Sequencing of the Two Major Developmental Stages

Samples of the two major fungal developmental stages (mycelium and fruiting body) from the *G. incarnatum* strain CCMJ2665 were provided by the mushroom section of the Engineering Research Center of the Chinese Ministry of Education for Edible and Medicinal Fungi, Jilin Agricultural University (Changchun, China). RNA extraction and quality control were performed following the processes of Fu et al. [16]. cDNA libraries were constructed, and 150 paired-end sequencing was performed on an Illumina HiSeq 4000 platform at Novogene Co., LTD (Beijing, China). Sequencing data have been deposited in the NCBI SRA (accession no. PRJNA510218).

Raw data were filtered to remove adapter sequences and low-quality reads for downstream analyses. The trimmed reads were mapped to the *G. incarnatum* genome using TopHat v2.0.12 [39]. The number of reads mapped to each gene was counted using HTSeq v0.6.1 [40]. Fragments per kilobase of transcript per million mapped reads (FPKM) values were used to calculate gene expression. Genes differentially expressed between developmental stages were identified using the DESeq package (1.18.0) [41] in R with adjusted *p*-value set to <0.05.

## 3. Results and Discussion

### 3.1. Genome Sequencing and Assembly

**Figure 1:**
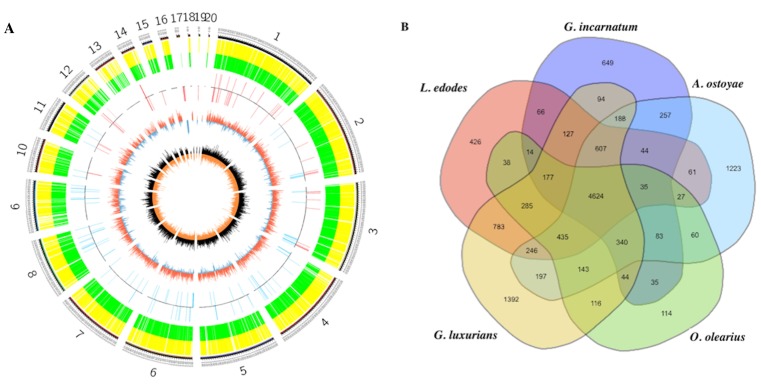
The *Gloeostereum incarnatum* genome and comparative genomics analysis. (**A**) The *G. incarnatum* genome. Outside to inside of concentric circles show assembly scaffold number, gene density, non-coding RNA (ncRNA), GC count and GC skew. (**B**) Unique and homologous gene families. The number of unique and shared gene families is shown in each of the diagram components and the total number of gene families for each fungus is given in parentheses.

A high-quality reference genome for *G. incarnatum* was generated from a protoplast monokaryon isolated from the dikaryotic strain of a commercial *G. incarnatum* cultivar (CCMJ2665; see Table 1). The genomic DNA of *G. incarnatum* was sequenced on PacBio SMRT Sequel platform generating ~94× coverage of 3,642 Mbp of clean data, as shown in Table S1. Compared to other edible and medicinal mushrooms, the assembled genome of *G. incarnatum* (38.7 Mbp), as shown in Figure 1, was of an intermediate size; the *Wolfiporia cocos* genome was the largest (50.5 Mbp); and the *Agaricus bisporus* var. *bisporus* genome was the smallest (30.2 Mbp), as shown in Table 1 [11,12,13,42,43,44,45,46,47,48]. *G. incarnatum* had a guanine-cytosine (GC) content of 49%; GC content in the other mushroom genomes examined was 45.3–55.9% (Table 1). The genome of *G. incarnatum* was one of the most complete assembled genomes across all representative edible and medicinal mushrooms examined, consisting of 20 scaffolds with an N50 of 3.5 Mbp (Table 1; Figure 1). The completeness of the *G. incarnatum* genome assembly was analyzed with the CEGMA [17] and the single-copy orthologs test using Fungi BUSCOs [18]. The CEGMA analysis indicated that 96.8% of the core eukaryotic genes were mapped to the *G. incarnatum* genome. The BUSCO analysis suggested that the annotation set was well completed, with 93.1% complete BUSCOs and 4.5% missing BUSCOs. Thus, our results indicate that the *G. incarnatum* genome assembly is high quality.

### 3.2. Gene and Repeat Sequence Prediction and Annotation

To most accurately predict the protein-coding genes in the *G. incarnatum* genome, we used a homology-based prediction strategy (against four representative mushroom genomes) combined with de novo gene prediction approaches. We predicted 15,251 protein-coding genes, accounting for 57.46% of the assembled *G. incarnatum* genome (Table S2). The predicted protein-coding genes had an average length of 1456.86 bp and contained 4.38 exons (each with an average length of 264.46 bp). The protein-coding genes were functionally annotated against several databases: NCBI nr, Swiss-Prot, InterPro, GO, COG, and KEGG. Of the 15,251 protein-coding genes predicted, 72.62% had homologs in one or more of the databases searched (Table S2).

We identified ~5.9 Mbp of repeat sequences in the *G. incarnatum* genome. Of these repeat sequences, 0.49% were predicted to be tandem repeats and 14.46% to be transposons (TEs) (Figure 1; Table S3). Most of the predicted TEs were long terminal repeats (LTRs), representing 13.78% of the genome (Table S3). Of the non-coding RNA species we identified in the *G. incarnatum* genome, 161 were tRNAs and 44 were rRNAs (Table S4). Nine of the identified tRNAs were possible pseudogenes, and the remaining 152 anti-codon tRNAs corresponded to the 20 common amino acids (Table S4). We also predicted 18 miRNAs and 18 snRNAs; the snRNAs comprised 15 spliceosomal RNAs and three C/D box small nucleolar RNAs (Table S4).

KOG functionally classified 4243 (27.82%) of the predicted proteins. Of these, 499 genes were associated with “amino acid transport and metabolism”, 476 genes were associated with “carbohydrate transport and metabolism”, and 337 with “secondary metabolite biosynthesis, transport, and catabolism”. This suggests that several *G. incarnatum* proteins are involved in nutrient absorption, transformation, and the synthesis of secondary metabolites. Similarly, KEGG classification indicated that both “amino acid metabolism” and “carbohydrate metabolism” were enriched in *G. incarnatum* genes (603 and 654 genes, respectively). KEGG analysis indicated that another 161 proteins were assigned to “biosynthesis of other secondary metabolites”, and 59 proteins were associated with the “metabolism of terpenoids and polyketides”. As the medicinal properties of edible mushrooms are closely related to the biosynthesis of secondary metabolites [49], these compounds were the focus of the remainder of our study.

### 3.3. Comparative Genomics and Evolutionary Analysis

With the exception of the *G. incarnatum* genome assembled in this study, no complete genomes are available for other fungi in Cyphellaceae. Thus, our evolutionary analysis compared whole genome sequences of seven representative species of the Agaricales: *O. olearius*, *G. luxurians*, *L. bicolor*, *C. cinerea*, *A. ostoyae*, *L. edodes*, and *S. commune*. We also included genomes of two additional agaricomycetid species having fossil calibrations—*S. lacrymans* and *C. puteana* [50]. We found that the *G. incarnatum* genome includes 7384 gene families, with 10,075 (66.1%) genes having homologs in at least one of the other nine fungal species (Figure 1, Table S5). Interestingly, 5,176 (33.9%) unclustered genes and 469 unique gene families (containing 1369 genes) were *G. incarnatum* specific (Table S5). These *G. incarnatum*-specific genes were associated with diverse biological processes, including steroid biosynthesis, terpenoid backbone biosynthesis, and polysaccharide biosynthesis.

**Figure 2:**
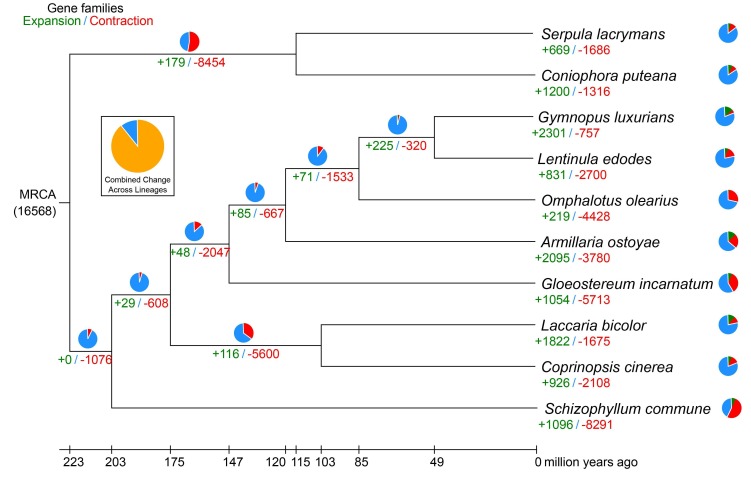
The *Gloeostereum incarnatum* genome evolutionary analysis. The number of expanded (green) and contracted (red) gene families is shown at each branch. The estimated divergence time (MYA: million years ago) is shown at the bottom. MRCA: most recent common ancestor.

We then constructed an ML phylogeny for *G. incarnatum* and the nine additional fungal species, based on 1822 shared single-copy orthologous genes (Figure 2). These data indicate that *G. incarnatum* is phylogenetically closer to *A. ostoyae*, diverging ~174 million years ago (Figure 2). We also identified 325 significantly expanded gene families in the *G. incarnatum* genome (*p* ≤ 0.01) (Figure 2); these families were primarily associated with carbohydrate metabolism (starch/sucrose metabolism and glycolysis/gluconeogenesis), amino acid and lipid metabolism, and genetic and environmental information processing. However, caution is warranted when interpreting species’ evolutionary time estimates and gene family expansions and contractions based on genomes generated with different sequencing platforms, assembly methods, and selection of comparative analysis groups. Nevertheless, our analysis provides new insights into the phylogeny of *G. incarnatum* and other mushroom species based on whole-genome data.

### 3.4. The Decomposition of Wood by CAZymes

**Figure 3:**
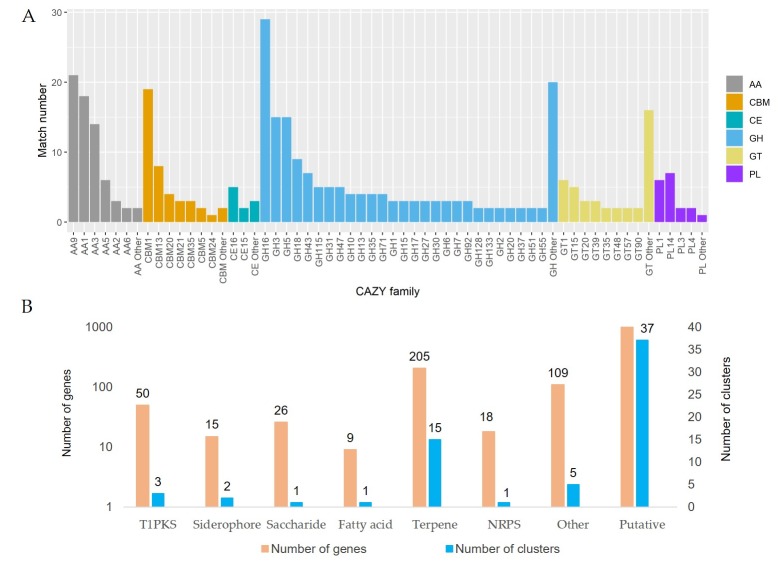
(**A**) Annotation of carbohydrate-related genes in the *G. incarnatum* genome; (**B**) secondary metabolite-related gene clusters in the *G. incarnatum* genome. T1PK: Type I polyketide synthases; NRPS: nonribosomal peptide synthetase.

To further classify the proteins associated with lignin digestion during carbohydrate metabolism, we mapped the protein sequences of *G. incarnatum* to the CAZy database [36]. We identified 311 non-overlapping CAZymes in six families in *G. incarnatum* (Table S6); the CAZymes in *G. incarnatum* were more diverse and abundant than those of brown rot fungi [51]. The *G. incarnatum* CAZymes consisted of 164 GHs, 66 proteins with AAs, 42 CBMs, 41 GTs, 18 PLs, and 10 CEs (Figure 3).

As the GHs include many cellulase families (such as GH16, GH5, GH3, GH6, and GH7) [36], the remarkably higher number of GHs in *G. incarnatum* was not unexpected. As a saprotrophic mushroom, *G. incarnatum* is likely to require many GHs to decompose cellulose from its woody hosts. AAs were the next most abundant family of CAZymes in *G. incarnatum*; AAs identified in this species included 21 AA9, 18 AA1, and 14 AA3 enzyme families. These three AA families are also the most abundant AAs in other fungi [10,11,12,13]. However, only three AA2 family proteins, the lignin-modifying fungal peroxidases (PODs), were identified in *G. incarnatum*. PODs are the primary lignin decomposers in the model white rot fungus *Phanerochaete chrysosporium* and other fungal species [52]. As *G. incarnatum* is restricted to elm tree hosts, the few AA2s identified in this fungus may be sufficient to decompose lignins produced by elm. We also identified 21 genes encoding enzymes for pectin digestion in *G. incarnatum*. Thus, the wood-decaying mushroom, *G. incarnatum*, may utilize complex strategies to decompose plant cell walls.

### 3.5. Secondary Metabolites and Terpene Pathway

The pharmacological properties of medicinal mushrooms are largely conferred by secondary metabolites; these metabolites have received intense research attention [1,49,53]. Here, we used antiSMASH to search for gene clusters encoding secondary metabolites in *G. incarnatum* [38]. We identified 65 gene clusters: one saccharide, 15 terpene synthases (TSs), one fatty acid, three polyketide synthases (PKSs), two siderophores, one non-ribosomal peptide-synthetase (NRPS), and 37 putative gene clusters of unknown type (Figure 3).

**Figure 4:**
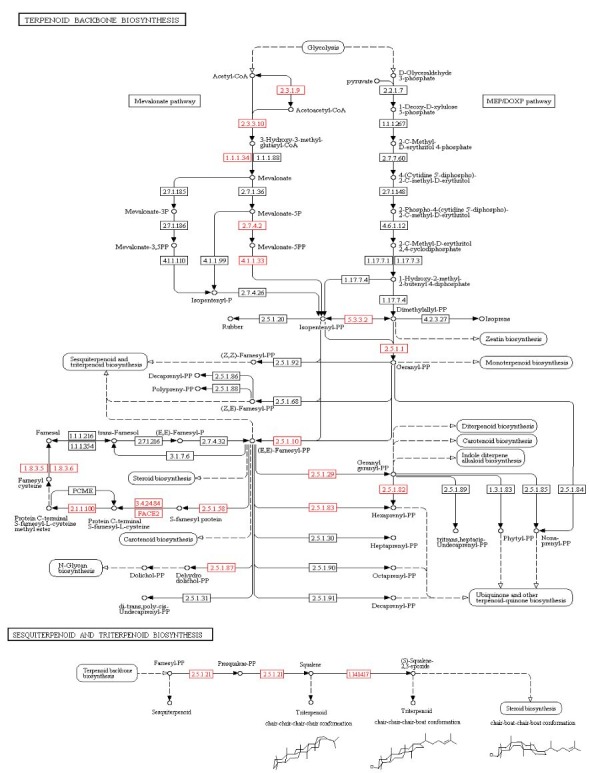
“Terpenoid backbone biosynthesis” (KEGG: ko00900) and “sesquiterpenoid and triterpenoid biosynthesis” (KEGG: ko00909) pathways of *G. incarnatum*. Red boxes indicate the presence of the enzymes, whereas white boxes indicate enzyme is not present.

Terpenoid biosynthesis is of particular interest as terpenoids are important pharmacologically active compounds in *G. incarnatum* [14,54]. *G. incarnatum* contains two unique sesquiterpene compounds, gloeosteretriol and incarnal [14,54]. Both compounds demonstrate antibacterial activity against the Gram-positive bacteria *Staphylococcus aureus* and *Bacillus subtilis*, but not against any of the Gram-negative bacteria tested to date [14,54]. Incarnal extracted from *G. incarnatum* and another fungus in Cyphellaceae (*Chondrostereum* sp.) also shows potent cytotoxicity against several cancer cell lines [8,55]. To further investigate the biosynthesis of terpenoids in *G. incarnatum*, we mapped 35 proteins to 17 enzymes in the “terpenoid backbone biosynthesis” (KEGG: ko00900) pathway, and 10 proteins to four enzymes in the “sesquiterpenoid and triterpenoid biosynthesis” (KEGG: ko00909) pathway (Figure 4). The pathway mapping results suggested that the *G. incarnatum* terpenoids are likely to be synthesized through the mevalonate (MVA) pathway, not the 2-C-methyl-D-erythritol 4-phosphate/1-deoxy-d-xylulose 5-phosphate (MEP/DOXP) pathway. This is in line with the results for other mushrooms, such as *G. lucidum* and *H. erinaceus* [11,12].

Sesquiterpenoids are synthesized from farnesyl diphosphate (FPP) by various sesquiterpene synthases [56]. We located five genes encoding sesquiterpene synthases in *G. incarnatum*—three encoding trichodiene synthase (EC 4.2.3.6) and two encoding aristolochene synthase (EC 4.2.3.9; Figure 4). Interestingly, trichodiene is a precursor for the biosynthesis of the mycotoxin nivalenol, which is widely found in *Fusarium* species, and the biosynthesis of aristolochene, which is a precursor for the PR toxin found in *Penicillium* species [57,58]. To the best of our knowledge, neither of these compounds are produced by *G. incarnatum*. It would be interesting to know if trichodiene or aristolochene was a substrate for the synthesis of gloeosteretriol or incarnal in *G. incarnatum*. Based on structural similarity, incarnal might potentially be synthesized from trichodiene in conjunction with certain cyclization, bond-shift rearrangement, oxidation, and hydroxylation reactions. Further experiments are thus necessary to confirm the production of trichodiene and aristolochene, as well as their association with the biosynthesis of gloeosteretriol or incarnal, in *G. incarnatum*.

Regarding triterpenoid, two farnesyltransferases (EC 2.5.1.21), three squalene monooxygenases (EC 1.14.14.17), and one lanosterol synthase (EC 5.4.99.7) were encoded in the *G. incarnatum* genome. This suggested that *G. incarnatum* synthesizes squalene, (S)-2,3-epoxysqualene, and lanosterol, all of which are intermediates in the synthesis of triterpenoid and sterol [59]. Notably, lanosterane-type triterpenoids are produced by several medical mushrooms, including species of *Ganoderma*, *Innonotus*, and *Antrodia* (reviewed in [60]), although the relevant biosynthesis pathways are unknown. The existence of these triterpenoid-related proteins in *G. incarnatum* suggests that this species may produce previously uncharacterized triterpenoids.

### 3.6. The CYP Family

Although the pathway for terpenoid backbone biosynthesis in fungi is relatively well studied [61,62], the steps following terpenoid cyclization are largely unknown. The structural diversity of terpenoids depends on post-modification of many specific chemical groups. These modifications involve a series of hydroxylation, reduction, oxidation, and acylation reactions, largely mediated by CYPs (cytochrome P450s) [63,64,65]. In fungi, CYPs are especially important for xenobiotic degradation and the biosynthesis of several secondary metabolites, including terpenoids and polyketides [63]. Based on a comparative search of the Fungal Cytochrome P450 Database [66], 145 CYP proteins were identified in *G. incarnatum*. These proteins were classified into 57 families following Nelson’s nomenclature [37]. The family CYP5144 included the greatest number of *G. incarnatum* CYPs (16); CYP5144 also included the most CYPs in another medicinal mushroom, *Lignosus rhinocerotis* [46] (Table 2). It is thus likely that CYP5144 family proteins play key roles in the biosynthesis of terpenoids in *G. incarnatum*.

**Figure 5:**
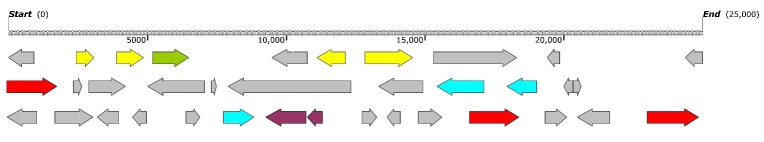
Genetic structures of sesterterpenoid synthase genes and their neighboring genes. Each gene is represented by an arrow. The aristolochene synthase gene (GI_10003231) is indicated by green color; the trichodiene synthase genes (GI_10004653, GI_10004654 and GI_10004694) are indicated by light blue color; the cytochrome P450 (CYP) genes are indicated by red color; choline dehydrogenase genes are indicated by yellow color; The Sec1-like protein genes are indicated by purple color.

As previously noted, the *G. incarnatum* genome encoded two sesterterpenoid synthases—aristolochene synthase and trichodiene synthase. Interestingly, the trichodiene synthase genes (GI_10004653, GI_10004654, and GI_10004694), but not the aristolochene synthase gene, (GI_10003231) were identified in gene clusters containing several CYPs (i.e., CYP65X, CYP530A, and CYP617B; Figure 5). Based on the logic in the Fungal Cytochrome P450 Database (FDPD) pipeline [66], CYP530A and CYP617B were assigned to the families CYP512 and CYP5144, respectively. Both of these families may be involved in the biosynthesis of bioactive terpenoids in *G. lucidum* and *L. rhinocerotis* [12]. These results further support our hypothesis that incarnal, the bioactive sesterterpenoid produced by *G. incarnatum*, might be synthesized from trichodiene, mediated by CYPs. The second-largest family of CYPs identified in *G. incarnatum* was CYP620 (Table 2), which is a relatively rare family in other medicinal mushrooms (absent in *G. lucidum*, one in *A. cinnamomea*, and three in *L. rhinocerotis*) [11,12,46]. This indicates that CYP distributions and functions vary among medicinal mushrooms. The exact roles of the identified CYPs in terpenoid post-modification or other biological functions remain to be experimentally validated.

### 3.7. Polysaccharide Biosynthesis

Composition of *G. incarnatum* polysaccharides also had immunomodulatory and immuno-enhancing effects in a mice model [6]. Some of the most potent immunomodulatory polysaccharides produced by medical mushrooms are water soluble 1,3-β- and 1,6-β-glucans [67]. In *G. incarnatum*, we identified four 1,3-β-glucan synthases (K00706 and K01180), three UTP–glucose-1-phosphate uridylyltransferases (K00963), 12 GTPase-activating-associated proteins (K12492, K12493, K19838, K19844, K19845, K14319, K17265, K18470, K20315, and K19839), two hexokinases (K00844), and two phosphoglucomutases (K01835) (Table 3). We also identified 15 β-glucan biosynthesis-associated proteins (PF03935; Table 3); β-glucan biosynthesis-associated proteins were shown to be involved in the biosynthesis of 1,6-β-glucans in *Saccharomyces cerevisiae* [68]. The polysaccharide biosynthesis-related proteins identified in *G. incarnatum* are summarized in Table S5. Compared with five other species of medicinal mushrooms (*Auricularia heimuer* [69], *A. cinnamomea* [11], *Sparassis latifolia* [47], *L. rhinocerotis* [46], and *G. lucidum* [12]), *G. incarnatum* produced more 1,3-β-glucan synthases, GTPase-activating-associated proteins, and β-glucan biosynthesis-associated proteins, as well as similar numbers of UTP–glucose-1-phosphate uridylyltransferases, hexokinases, and phosphoglucomutases (Table S7). This suggests that *G. incarnatum* might produce more 1,3-β- and 1,6-β-glucans. In-parallel quantifications of 1,3-β- and 1,6-β-glucan production among these medicinal mushrooms during different growth phases should be performed and compared.

### 3.8. Transcriptomic Analysis

**Figure 6:**
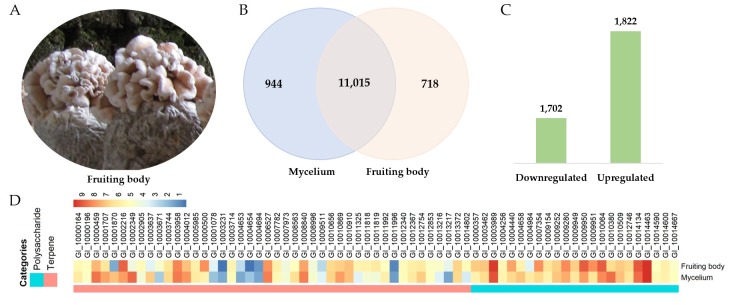
Comparative transcriptome profiling of the mycelium and the fruiting body of *G. incarnatum*: (**A**) Fruiting bodies of *G. incarnatum*; (**B**) Venn diagram of the genes expressed in the mycelium and/or the fruiting body; (**C**) number of genes being significantly downregulated or upregulated in the fruiting body compared with the mycelium; (**D**) heatmap of the genes associated with the biosynthesis of polysaccharides and terpenes.

As the expression levels of target genes encoding pharmacologically relevant proteins in *G. incarnatum* might differ across developmental stages, we profiled the transcriptomes of two major developmental stages of *G. incarnatum*—the mycelium and the fruiting body. We generated 70,634,952 raw reads from the cDNA libraries of the two stages. After data filtering and trimming, 69,716,944 high-quality clean reads remained. Of these clean reads, 92% were successfully mapped to the *G. incarnatum* genome. Across both stages, 11,015 genes were expressed, with 944 genes expressed only in the mycelium, and 718 genes only expressed in the fruiting body (Figure 6). We identified 3524 differentially expressed genes (DEGs) in the fruiting body as compared to the mycelium (Figure 6). Of these 1822 were significantly upregulated in the fruiting body as compared to the mycelium, and 1702 were significantly downregulated (Figure 6).

The gene expression patterns in the mycelium and fruiting body of *G. incarnatum* varied depending on the type of secondary metabolite encoded. For example, 17 of 45 terpenoid biosynthesis-related genes (38%) were differentially expressed between the mycelium and the fruiting body (Figure 6, Table S8). Of these, 65% were upregulated in the mycelium as compared to the fruiting body (Figure 6, Table S8), indicating that the biosynthesis of terpenoid compounds might be greater in the mycelium of *G. incarnatum*. In contrast, 10 of the 23 genes associated with polysaccharide biosynthesis (43%) were differentially expressed between the mycelium and the fruiting body, with 70% of these being significantly upregulated in the fruiting body as compared to the mycelium (Figure 6). This indicates that the fruiting body of *G. incarnatum* might be a richer source of polysaccharides. These findings were consistent with those for terpenoid- and polysaccharide-related genes in *H. erinaceus* [13]. Therefore, different secondary metabolites might be more enriched at different fungal development stages. Due to the complexity of secondary metabolite biosynthesis, further studies should investigate the molecular mechanisms underlying the secondary metabolism of *G. incarnatum*.

## 4. Conclusions

In this study, we presented the first whole-genome sequence of *G. incarnatum*, which is the first sequenced genome for a fungus belonging to Cyphellaceae. The *G. incarnatum* genome is one of the most completely assembled edible mushroom genomes available to date, consisting of 20 scaffolds with an N50 of 3.5 Mbp. The remarkably higher number of GHs and AAs in *G. incarnatum* may contribute to the active decomposition of lignin and cellulose of its woody hosts. We identified 65 gene clusters involved in the biosynthesis of secondary metabolites in the *G. incarnatum* genome. We also investigated the functions of the proteins involved in terpenoid biosynthesis; terpenoids are one of the main types of pharmacologically active compounds produced by *G. incarnatum*. We found two sesquiterpenoid synthase genes, one encoding aristolochene synthase and the other encoding trichodiene synthase, in gene clusters enriched with CYP genes. This suggested that CYPs play an active role in the post-modification of aristolochene and trichodiene sesquiterpenoids. We also predicted 38 proteins involved in polysaccharides biosynthesis, another main class of bioactive compounds in *G. incarnatum*. Genes involved in terpenoid biosynthesis were generally upregulated in mycelium, while the polysaccharide biosynthesis-related genes were upregulated in the fruiting body. These results provide a foundation for future studies of the genetic basis underlying the medicinal properties of *G. incarnatum*.

## Figures and Tables

**Table 1:** Comparison of genome assembly among representative edible mushrooms.

Organism	Accession	Genome Size (Mbp)	Genome	Scaffold	N50 (Kbp)	GC Content (%)	Protein-Coding Genes	Sequencing Method
*Gloeostereum incarnatum*		38.7	94×	20	3500	49.0	15,251	PacBio Sequel
*Lentinula edodes*	LSDU00000000	46.1	60×	31	3663	45.3	13,426	PacBio RSII; Illumina HiSeq 2500
*Agrocybe aegerita*	PRJEB21917	44.8	253×	122	768	49.2	14,113	PacBio RSII; Illumina HiSeq 2500
*Hericium erinaceus*	PRJN361338	39.4	200×	519	538	53.1	9895	Illumina MiSeq; Hiseq 2500
*Antrodia cinnamomea*	JNBV00000000	32.2	878×	360	1035	50.6	9254	Roche 454; Illumina GAIIx
*Ganoderma lucidum*	AGAX00000000	43.3	440×	82	1388	55.9	16,113	Roche 454; Illumina GAII
*Wolfiporia cocos*	AEHD00000000	50.5	40×	348	2539	52.2	12,212	Sanger; Roche 454
*Inonotus baumii*	LNZH00000000	31.6	186×	217	267	47.6	8455	Illumina HiSeq
*Agaricus bisporus* var. *bisporus*	AEOK00000000	30.2	8.3×	29	2300	46.6	10,438	Sanger
*Lignosus rhinocerotis*	AXZM00000000	34.3	180×	1338	90	53.7	10,742	Illumina Hiseq 2000
*Sparassis latifolia*	LWKX00000000	48.1	601×	472	641	51.4	12,471	Illumina HiSeq 2500
*Flammulina velutipes*	BDAN00000000	35.3	132×	5130	150	49.6	13,843	Illumina HiSeq 2500

**Table 2:** Summary of the CYP genes in the *G. incarnatum* genome.

Family	Subfamily	Corresponding Gene Number	Total Gene Number	Family	Subfamily	Corresponding Gene Number	Total Gene Number
CYP5144	C,F	15,1	16	CYP675	A	3	3
CYP620	A,B,E,H	1,1,4,2	8	CYP682	B	3	3
CYP5015	C	6	6	CYP504	A	3	3
CYP5014	F,H	2,3	5	CYP51	F	3	3
CYP5068	B	5	5	CYP55	A	3	3
CYP5080	B,D	3,2	5	CYP65	J,X	1,1	2
CYP5093	A	5	5	CYP5070	A	2	2
CYP505	C,D	3,1	4	CYP5074	A	2	2
CYP535	A	4	4	CYP5078	A	2	2
CYP536	A	4	4	CYP5081	A	2	2
CYP617	A,B	1,2	3	CYP5125	A	2	2
CYP5037	B	3	3	CYP540	B	2	2
CYP5110	A	3	3	CYP630	B	2	2
CYP530	A	3	3	Others	-	-	30

**Table 3:** Summary of the polysaccharide biosynthesis-related proteins in *G. incarnatum*.

Enzyme Family	KO Term	EC Number	Gene Number	Gene Name
1,3-β-glucan synthase	K01180	EC:3.2.1.6	1	GI_10004256
	K00706	EC:2.4.1.34	3	GI_10014134, GI_10014600, GI_10010064
UTP–glucose-1-phosphate uridylyltransferase	K00963	EC:2.7.7.9	3	GI_10009949, GI_10009950, GI_10009951
Hexokinase	K00844	EC:2.7.1.1	2	GI_10010509, GI_10009252
Phosphoglucomutase	K01835	EC:5.4.2.2	2	GI_10003989, GI_10014463
GTPase-activating-associated protein	K12492	-	1	GI_10009154
	K19838	-	1	GI_10009280
	K12493	-	1	GI_10004440
	K14319	-	1	GI_10004658
	K19845	-	2	GI_10004984, GI_10007354
	K19839	-	3	GI_10003462, GI_10010380, GI_10012746
	K19844	-	2	GI_10014590, GI_10000357
	K18470	-	1	GI_10014667

## Supplementary Materials

Supplementary materials can be found at https://www.mdpi.com/2073-4425/10/3/188/s1.

## Abbreviations

SMRT	Single-Molecule, Real-Time
KEGG	Kyoto Encyclopedia of Genes and Genomes
CYP	cytochrome P450
CAZymes	carbohydrate-active enzymes
DEG	differentially expressed genes
FPKM	fragments per kilobase of transcript per million mapped reads
